# Copy Number Variation and Rearrangements Assessment in Cancer: Comparison of Droplet Digital PCR with the Current Approaches

**DOI:** 10.3390/ijms22094732

**Published:** 2021-04-29

**Authors:** Vincenza Ylenia Cusenza, Alessandra Bisagni, Monia Rinaldini, Chiara Cattani, Raffaele Frazzi

**Affiliations:** 1 Laboratory of Translational Research, Azienda Unità Sanitaria Locale—IRCCS di Reggio Emilia, 42122 Reggio Emilia, Italy; VincenzaYlenia.Cusenza@ausl.re.it; 2 Pathology Unit, Azienda Unità Sanitaria Locale—IRCCS di Reggio Emilia, 42122 Reggio Emilia, Italy; Alessandra.Bisagni@ausl.re.it; 3 Medical Genetics Unit, Azienda Unità Sanitaria Locale—IRCCS di Reggio Emilia, 42122 Reggio Emilia, Italy; Monia.Rinaldini@ausl.re.it (M.R.); Chiara.Cattani@ausl.re.it (C.C.)

**Keywords:** droplet digital PCR, FISH, MLPA, copy number variation assessment

## Abstract

The cytogenetic and molecular assessment of deletions, amplifications and rearrangements are key aspects in the diagnosis and therapy of cancer. Not only the initial evaluation and classification of the disease, but also the follow-up of the tumor rely on these laboratory approaches. The therapeutic choice can be guided by the results of the laboratory testing. Genetic deletions and/or amplifications directly affect the susceptibility or the resistance to specific therapies. In an era of personalized medicine, the correct and reliable molecular characterization of the disease, also during the therapeutic path, acquires a pivotal role. Molecular assays like multiplex ligation-dependent probe amplification and droplet digital PCR represent exceptional tools for a sensitive and reliable detection of genetic alterations and deserve a role in molecular oncology. In this manuscript we provide a technical comparison of these two approaches with the golden standard represented by fluorescence in situ hybridization. We also describe some relevant targets currently evaluated with these techniques in solid and hematologic tumors.

## 1. Introduction

Fluorescence in situ hybridization (FISH) represents the reference method for gene deletion and amplification assessment. Today, this approach and its variants are still the gold standard in many genetics laboratories dealing with deletions and amplifications or rearrangements [1,2].

It is an informative and versatile technique that has been applied to the study of tumor protein 53 (*TP53*), human epidermal growth factor receptor 2 (*HER2*), anaplastic lymphoma kinase (*ALK*), baculoviral IAP repeat containing 3 (*BIRC3*), rearranged during transfection (*RET*) and many other targets during the years [1,3,4,5,6,7].

FISH presents some limitations and caveats though, first of all the relatively low sensitivity. Only large gene deletions or amplifications can be detected and quantified by the probes by immunofluorescence. Furthermore, FISH does not provide information on the fusion partner (when investigating gene rearrangements) and has been shown to be limited in specificity for common targets such as *ALK* [2]. The technique also bears the variability due to manual preparation of glasses and to the microscopy skills of the geneticists.

In recent years, some molecular techniques have been developed and tested successfully with the purpose of improving the copy number variation (CNV) detection and quantitation. Multiplex ligation-dependent probe amplification (MLPA), droplet digital PCR (ddPCR) and next-generation sequencing (NGS) encompass these techniques.

MLPA is an accurate and time-efficient approach to detect genomics deletions and insertions, which are frequently causes of cancers. MLPA can successfully detect the CNV of all exons within a gene simultaneously and with high sensitivity. Besides the detection of CNV and rearrangement assessments in cancers and other diseases, another application of MLPA is the analysis of the ploidy in human cells [8]. ddPCR is an innovative technique, mainly used for research purposes. This technique was developed to provide high-precision and absolute quantitation of nucleic acid target sequences. Several are the emerging application of ddPCR, such as: absolute quantitation, CNVs, detection of rare sequence, gene expression, microRNA analysis, library quantitation for next-generation sequencing (NGS) and genome editing detection (www.bio.rad.com (accessed on 1 April 2021)).

Targeted next-generation sequencing approaches (NGS) have proven to be affective in the detection of chromosomal aberrations like translocation of *ALK* and lysine methyltransferase 2A (*KMT2A*) in lung carcinoma, anaplastic large cell carcinoma and acute leukemias [9]. Sensitivity and specificity resulted to be similar to routine FISH with the advantage of single nucleotide breakpoint resolution and the well known capabilities of deep-sequencing to discover new variants. Furthermore, the NGS method demonstrated to have a very low rate of false positive detections [9]. NGS has been used also for the detection of hereditary deletions, for instance, of genes related to CRC [10].

However, some limits can be represented by variability in tumor cellularity, input DNA levels and sequencing coverage. Last but not least, NGS requires dedicated bioinformatics platforms and skills, which not all the routine laboratories can afford. Thus, next generation sequencing approaches deserve a dedicated review [9,11].

In the present manuscript, MLPA and ddPCR are reviewed. Relevant examples of solid and hematologic tumors like breast, lung and colon cancers in addition to chronic lymphocytic leukemia and gliomas are considered. The translational potential of ddPCR for the detection of copy number variations (CNVs) in several examples is reported and *EMAP* like 4-anaplastic lymphoma kinase-*ALK* (*EML4-ALK*) gene rearrangements in the molecular oncology field is commented.

## 2. Fluorescence in Situ Hybridization (FISH)

CNVs are notable sources of genetic variation in human DNA and result involved in a large number of diseases. For diagnostic purposes the main technique used in routine clinical laboratory is FISH [12,13,14].

FISH is an approach that uses fluorescently labeled DNA probes to detect chromosomal and cytogenetic alterations like aneusomy, duplication, amplification, deletion and translocation. There are four types of FISH probes: chromosome enumeration probes (CEPs), locus-specific indicator (LSI) probes, repetitive-sequence probes (RSPs) (centromeric and telomeric) and whole chromosome painting (WCP) [15].

**Figure 1:**
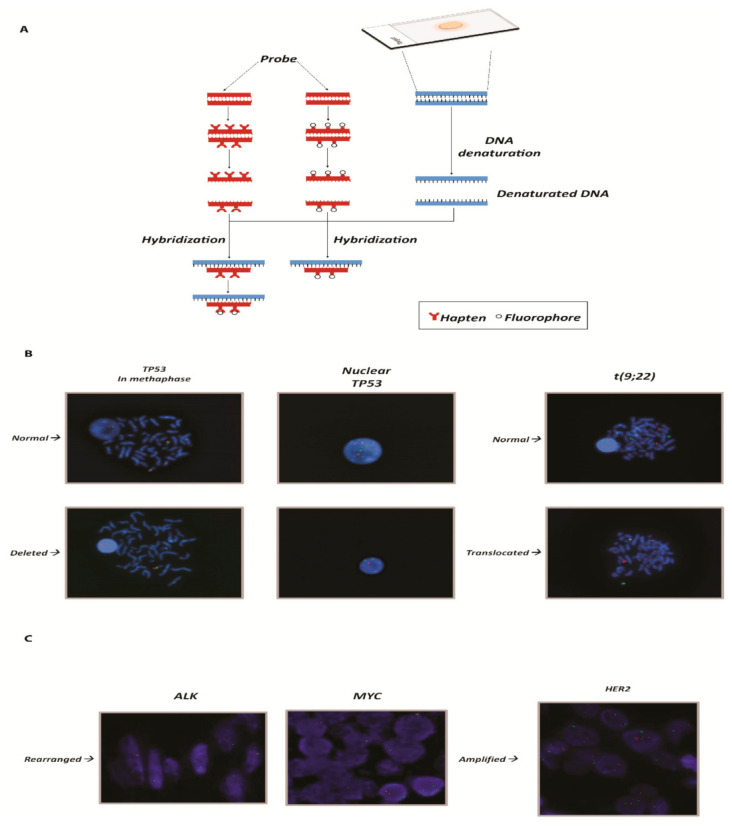
(**A**) Graphical representation of the FISH technique: from the adhesion of the nucleic acid to the hybridization of the probes. (**B**) On the left panels, example of a gene deleted in chronic lymphocytic leukemia. The slides show the *TP53* gene (17p13.1) represented by the red spot [cosmid probes for TP53 deletion (Cytocell)] and the control (centromere 17) by the green spot. In the normal nucleus and metaphase, the number of red spots is equal to the number of green spots and they are both diploid. In the deleted nucleus the number of red spots is equal to 1, instead of two. On the right panels, example of translocation in chronic myeloid leukemia. The translocation in question concerns the t(9;22) of the genes *ABL* (9q34.12) and *BCR* (22q11.21), known as the Philadelphia chromosome. In the upper slide a normal karyotype is represented. *BCR* and *ABL* are clearly distinguishable and highlighted by a dual fusion cosmid probes for BCR/ABL (ABL1) translocation. The lower slide shows the translocation: the two spots overlap and are displayed as a yellow spot. (**C**) Example of rearranged and amplified genes: on the left *ALK* and *MYC* genes, respectively, in cervical cancer and B-cell lymphoma. The rearrangement in FISH can be seen because the two spots, one that identifies the gene (red tag; LSI ALK dual color break apart probe (Vysis) and LSI MYC dual color break apart rearrangement probe (Vysis)) and one that identifies the centromere, which is used as a control (green tag), are distant. On the right panel, example of amplification of the *HER2* gene in breast cancer. The red spots (identified in the images by dual color probe LSI HER2 spectrum orange) that identify *HER2* gene are in a greater number compared to the green spots that identify the control, represented by centromere 17 (CEP17 Spectrum Green PathVysion Vysis). DAPI staining (blue) is used for the nuclei morphology in all the presented panels. The magnification is 63×.

FISH probes were first developed in the late 1970s [16,17,18]. Continuous improvements have been made since then. The latter involved the engineering of probes and protocols with the development of robotic platforms, multiple detection and greater sensitivity [19,20,21,22,23,24,25,26,27]. Continuous efforts have significantly improved the signal-to-noise ratio, sensitivity and reproducibility of FISH in various applications. However, little technical advancement has been achieved in simplifying the procedure. FISH remains a labor-intensive and time-consuming approach contemplating technically complex protocols despite the fact that robotic platforms have been developed at the state-of-the-art facilities [28,29]. The procedure is represented in Figure 1.

## 3. Multiplex Ligation-Dependent Probe Amplification (MLPA)

MLPA is a multiplex amplifiable probe hybridization (MAPH)-related method developed by Mrc-Holland and presented for the first time in 2002. MLPA was developed to bypass the immobilization of the nucleic acid to the membrane, like Southern blot, that is difficult to introduce in routine diagnostics [30].

MLPA are based on a simple quantitative PCR reaction and can be performed using standard laboratory equipment. MLPA probes are comprised of a pair of single-stranded DNA half-probes that need to be joined by ligation before PCR amplification can occur. MLPA have been designed to determine, in one assay, the quantity and the copy number, of a set of specific sequences in a sample of interest. This technique allows the detection of deletions and duplications in genomic DNA in relation to specific genetic diseases. To achieve this, the method works with genomically unique sequences that hybridize to one target sequence only. After ligation, bound probes are coamplified and quantified. To discriminate the individual probes the original protocols encompass size separation by capillary electrophoresis. A MLPA probe set is designed so that the length of each of its amplification products is unique. In a diploid genome, a deletion will result in a 50% signal reduction of one or more probes while a duplication results in a 50% gain of signal [31,32,33,34].

The main advantages of MLPA can be summarized as follows:

- It is a cost effective way to check for rearrangements, duplications and deletions;
- It can be applied to a large number of targets (high throughput);
- It can be performed on a large number of samples simultaneously;
- It is reproducible, easy to perform, and it is capable of detecting a low quantity of the target;
- It requires only 50 ng of human DNA, can distinguish sequences differing by a single nucleotide and can detect small copy number differences.

**Figure 2:**
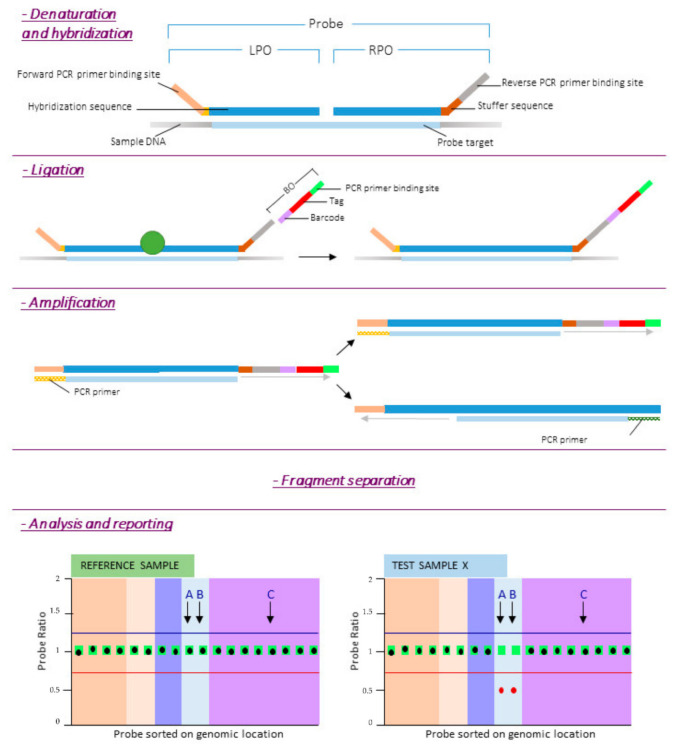
MLPA procedure. Reference DNA samples are required to determine the relative copy number of each of the probes. After denaturation of the target DNA, the left and right probe oligos (LPO and RPO respectively) are hybridized to their target sequence overnight (at least 16 h). The next day, the two oligonucleotides are enzymatically ligated and a barcode oligo (BO) is incorporated into the amplicon for sample identification. After PCR, products are separated by length. Finally quality check is performed and probe ratios calculated. A probe ratio of 1.0 corresponds to a normal diploid copy number, a probe ratio of 0.5 to a heterozygous deletion.

While being a very robust technique there are some limitations linked to MLPA. These limits are represented by inability to detect anomalies at the single cell level, inability to detect unknown point mutations, sensitivity to contaminants and to novel benign polymorphisms or to polymorphisms located near to a probe ligation site. The procedure is represented in Figure 2.

## 4. Droplet Digital PCR (ddPCR)

Droplet digital PCR is a robust technique that enables accurate absolute quantification of target molecules at a high degree of sensitivity. It essentially combines the simplicity of traditional end-point PCR and the quantification features of the real-time quantitative PCR (qPCR) methodologies. Unlike qPCR, the quantitation is absolute and does not utilize standards for calibration, leading to a faster, more precise and reproducible process [35,36].

The term “digital-PCR” was first used in 1999 to describe the application of the technique to the detection of *RAS* mutations by partitioning the sample. Digital PCR represents an evolution of the previously developed “limiting dilution PCR”. Digital PCR uses fluorescence instead of gel electrophoresis for the endpoint detection and, eventually, became widespread in the biomedical field due to technological improvements [37,38].

The basic principle of ddPCR involves an emulsion chemistry, which consists of creating emulsions of aqueous droplets in an oil medium where each droplet functions as a PCR unit [39,40]. A DNA sample is fractionated in a high number of droplets (up to 20,000). Each droplet contains all the reagents necessary for a PCR reaction, and basically functions as a micro-PCR reactor. If the droplet contains the template of interest, PCR amplification yields a positive signal. If there is no template, there is no signal [36]. The data are acquired and plotted through a two-channel fluorometer, allowing multiplexing of target genes, hence the term “digital” [36].

Minimum information for publication of quantitative digital PCR experiments guidelines (MIQE) were eventually published, with the aim of standardizing the experimental protocols. Specific MIQE for ddPCR (dMIQE) have been described and updated in recent papers in order to match the technological improvements [41,42].

**Figure 3:**
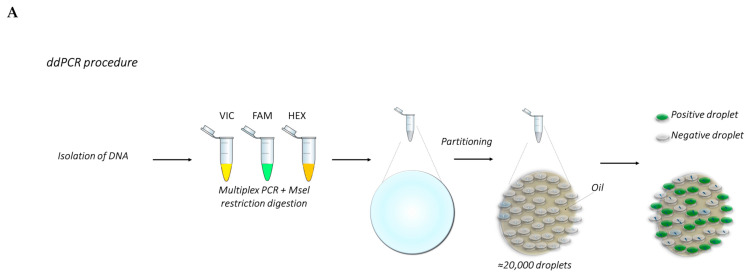

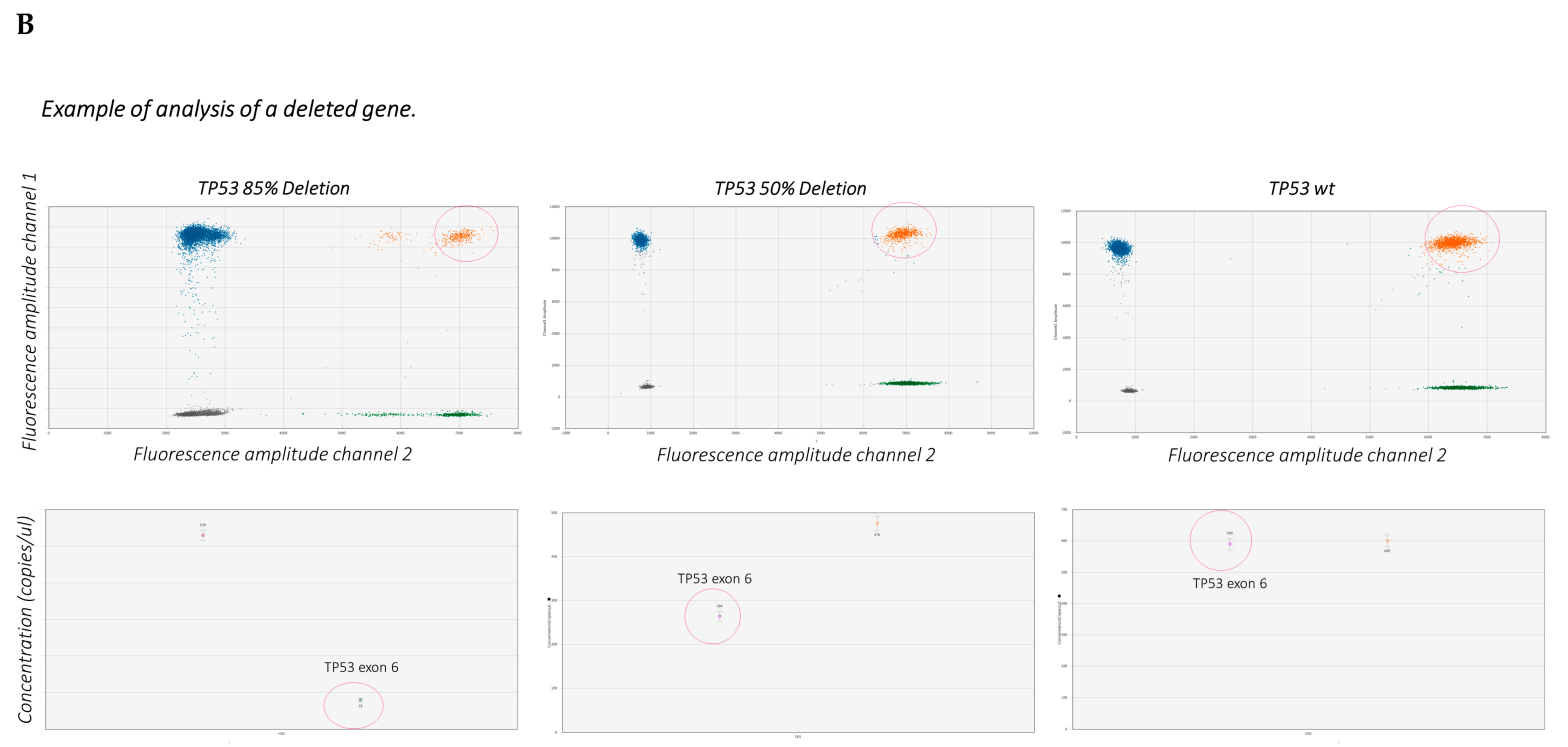
(**A**) Overview of the ddPCR procedure. (**B**) The graphs show examples of the analysis of TP53 CNV obtained from a previous work carried out by Frazzi and coauthors [43]. These graphs show the wild-type (wt) *TP53* gene (right panel), *TP53* with 50% deletion (middle panel) and 85% deletion (left panel). The increase in gene deletion corresponds to a decrease of the positive population (orange dots, red circled). The orange population is the double-positive one.

The most appreciated features of this system are:

- Reproducibility of the data, given by the absolute quantitation of the target genes;
- Sensitivity of the quantitation, obtained through the amplification into an emulsion matrix;
- Very low amount of starting material requested for any of the possible applications, due to the high sensitivity of the technique.
- The few disadvantages known up to now are:
- ddPCR requires special and dedicated instrumentation;
- ddPCR detects only known mutations.
- The procedure is represented in Figure 3.

## 5. Protocols and Graphical Representation of the Described Techniques

### 5.1. FISH Protocol 

#### 5.1.1. Slide Preparation

- Starting material is either formalin-fixed, paraffin-embedded tissue, needle aspirates or cell slides;
- Incubate with 200 µL RNase for 1 h at 37 °C
- Wash slides in 2× saline-sodium citate buffer (SSC) for 5 min, repeat.
- Rinse slides in 10 mM HCl.
- Incubate with 200 µL pepsin for 10 min at 37 °C.
- Rinse slides in deionized H_2_O.
- Wash slides in 2× SSC for 5 min, repeat.
- Stabilize slides in paraformaldehyde for 10 min.
- Wash slides in 2× SSC for 5 min, repeat.
- Dehydrate slides in an ethanol series: 70%, 80% and 95%; 2 min each.
- Air dry.

#### 5.1.2. Hybridization

- Prepare 30 µL hybridization solution per slide containing the specific probes for a target of interest. Heat to 70 °C for 10 min and place on ice.
- Place 30 µL of hybridization solution on each slide and cover with a plastic cover slip.
- Denature slide at 65–70 °C for 5 min on heat block.
- Gradually decrease temperature to 37 °C.
- Hybridize at 37 °C overnight in humidity chamber.

#### 5.1.3. Detection

- Wash slides in 2× SSC to remove coverslip.
- Wash slides in wash buffer at 40 °C for 5 min, repeat.
- Wash slides in 0.1× SSC at 40 °C for 5–15 min.
- Wash slides in 2× SSC at 40 °C for 5–15 min.
- Cool slides to room temperature.
- Equilibrate slides in detection buffer for 5 min.
- Block in blocking buffer for 20–30 min.
- Incubate with 50 µL antibody or detection compound for 30–60 min (e.g., 5 µg/mL Streptavidin-Cy3 in blocking buffer).
- Wash slides in 2× SSC for 5 min, repeat twice.
- Counterstain with DAPI solution for 10 min.
- Rinse briefly and mount in antifade mounting medium.
- Analyze with a fluorescence microscope.

### 5.2. MLPA Protocol

1. DNA denaturation:
  - Incubate 100 ng DNA sample in 5 μL of Tris-EDTA (pH 8) for 5 min at 98 °C.
2. Probes hybridization to sample DNA:
  - Cool down the samples to room temperature;
  - Add 3 μL hybridization master mix;
  - In a thermal cycler incubate 1 min at 95 °C followed by 16 h at 60 °C.
3. Ligation of hybridized probes:
  - Lower thermocycler temperature to 54 °C;
  - Add 32 μL Ligase-65 master mix, incubate 15 min at 54 °C;
  - Heat inactivates the ligase enzyme: 5 min at 98 °C.
4. PCR amplification of ligated probes:
  - Cool down the samples to room temperature;
  - Add 10 μL polymerase master mix at room temperature;
  Start PCR amplification.
5. Fragment separation by capillary electrophoresis and data analysis.

### 5.3. ddPCR Protocol

1. Prepare the samples by diluting genomic DNA at the desired concentration before setting up the reaction mix (e.g., dilute the DNA at 25 ng/microliter);
2. Prepare master mixes containing the specific reaction buffer for probes, each of the sampling and reference probes, 25 ng DNA/sample and water to a final volume of 20 μL/sample. The master mixes shall be sufficient for three-four technical replicates each target deletion;
3. Mix thoroughly and allow reaction tubes to equilibrate at room temperature for about 3 min;
4. Once the reaction mixtures are ready, load 20 μL of each reaction mix into a sample well of a cartridge, followed by 70 μL of droplet generation oil for probes into the oil wells;
5. Put the cartridge in the automated droplet generator;
6. After droplet generation, carefully transfer droplets into a clean 96-well PCR plate. Seal the plate with an aluminum foil in the PCR plate sealer.
7. Proceed to thermal cycling and PCR amplification, followed by the acquisition of droplets in the QX100 or QX200 droplet reader.
8. Design the experimental template through QuantaSoft™ Software (plate layout). After that the droplets acquisition is complete, data are analyzed with the proper setup for any specific application (CNV, gene expression, mutation detection, etc.).

**Figure 4:**
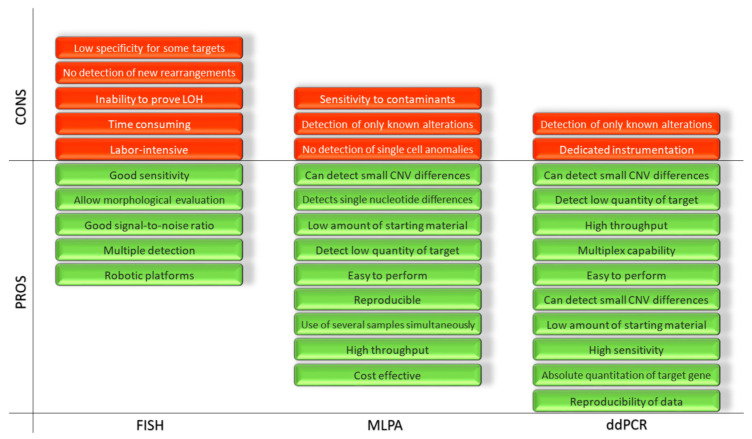
Representation of the advantages and disadvantages of the FISH, MLPA and ddPCR.

These three techniques, as shown, feature significant differences in terms of the number of steps, reagents and time required for the whole procedure. Therefore, they involve different resources in order to complete medical reports. A direct and schematic comparison is shown in Figure 4.

## 6. Time Required for the Report, Sensitivity and Specificity

A relevant and somehow overlooked issue is represented by the time required to produce a medical report. Regardless of whether the disease is acute or chronic, time is crucial and bears economics implications. The shorter the time required to produce a medical report, the less is the cost related to specialized personnel dedicated to the procedures.

In this regard, ddPCR is advantageous over FISH and MLPA when dealing with deletion- or duplication-detection. ddPCR is also very advantageous for the detection of gene rearrangements, useful during the differential diagnosis of different tumor types. In the recent paper by Frazzi R. and coworkers [43], the estimated time interval that is necessary from the moment of sample collection to the final, analyzed data is about 6 h. This time is comparable to that required by other molecular amplification techniques and is considerably shorter than that required by fluorescence microscopy-based techniques and multiplex ligation-dependent probe amplification. The required time ranges from about 15 h for FISH (starting to ready-made slides) to about 24 h for MLPA. Moreover, the number of ddPCR droplets counted by the fluorometer and used for data analysis constitute an objective measurement, at variance with the FISH positive foci that depend, to some extent, on the microscopist.

The analytical sensitivity and specificity of these three techniques is very high, according to the literature. FISH shows analytical specificity and sensitivity above 95%. MLPA is reported to have 95–99% of sensitivity and 93–97% of specificity whereas ddPCR above 98% for both [2,44,45,46].

## 7. Applications in Cancer Research and Molecular Testing

Cancer represents a major field of application for these three techniques, both for diagnostic and research purposes. To the present day, FISH and MLPA are mainly used for diagnostic purposes while ddPCR for research purposes.

Some of the most significant targets investigated by means of these three techniques in cancer are mentioned hereinafter (Table 1).

### 7.1. Chronic Lymphocytic Leukemia

*TP53* gene disruption is a major prognostic and predictive factor in chronic lymphocytic leukemia (CLL; [47]). It has been recently shown that ddPCR is an excellent approach to detect and quantitate *TP53* deletions in alternative to conventional FISH methods. According to the calculated diagnostic thresholds reported in the paper by Frazzi R. and coworkers, CLL patients with CNV values equal or above 1.83–1.90 are considered *TP53* non-deleted. On the contrary, patients having CNV values below these thresholds (calculated specifically for exons no. 5, 6 and 7) are deleted in one or more exons [43]. FISH and ddPCR demonstrate a high concordance for both non-deleted and deleted patients (93.1% and 90.0% respectively). Specificity and sensitivity result in being very high with this approach and the simultaneous probing for different exons ensures a higher percentage of detection in deleted patients.

Genomic aberrations of *TP53* can be also analyzed by MLPA. MLPA is an improvement of the polymerase chain reaction and can simultaneously detect several alterations like copy number changes, DNA methylation and point mutations of up to 50 genomic DNA sequences in a single experiment [48].

Previous studies have demonstrated that MLPA is a suitable technique to detect genomic aberrations in CLL and exhibits good correlation with FISH results [49,50]. Additionally, MLPA has the advantage of being faster and more cost-effective than FISH. While FISH provides information for only a limited number of genomic targets at the same time, MLPA can detect copy number alterations and point mutations simultaneously in multiple target regions [51].

Deletion 11q (del11q-) is another relevant karyotypic aberration in CLL associated to unfavorable prognosis [52]. Del11q is a recurrent karyotypic abnormality acquired by patients with progressive CLL disease. Initial karyotypic and FISH studies were complemented by genotypization of CLL patients, leading to the discovery that del11q is monoallelic, often large and includes a minimal deleted region encompassing ataxia telangiectasia mutated (*ATM*) gene and often also *BIRC3*, located on the 11q22.2 band, in cis with *ATM* [7,53].

*ATM* is involved in DNA damage repair whereas *BIRC3* is a negative regulator of non-canonical NF-kB signaling [53,54,55]. *BIRC3* deletion occurs in 83% of del11q cases and always coexists with *ATM* deletion, as demonstrated by the CLL4 study [53].

In the Italian study mentioned before, del11q- was investigated by FISH in the discovery cohort, where the deleted region included *BIRC3* in the 81.8% of cases. In the validation cohort both *ATM* and *BIRC3* were investigated by ddPCR, confirming the *ATM* deletion and showing the *BIRC3* deletion in 75.9% of the cases. The patients with a biallelic lesion of *BIRC3* (del and mut) showed a significant shorter time to first treatment when compared to *BIRC3*-del/wt or wild type patients [7].

*ATM* likewise *TP53* point mutations were investigated also by MLPA vs. FISH. These studies demonstrated that, although MLPA detected most recurrent copy number genomic aberrations (90.9%), false negative results were found in cases with small-size abnormal clones. False positive MLPA results may be obtained, instead, from samples with point mutations (*TP53*) or due to an apparent lack of probe specificity. Thus, MLPA may be a useful complementary but not alternative approach for FISH testing, in these cases [56].

*BIRC3* deletions and mutations are rare albeit unfavorable events for CLL patients that can be also associated to fludarabine-chemoresistance and to adverse prognosis in some chemotherapy-treated CLL patients [55,57]. Furthermore, a target resequencing of 22 genes of the patients enrolled in the UK LRF CLL4 study confirms that biallelic *BIRC3* lesions (del and mut) are an independent marker of inferior progression free survival (PFS) and overall survival (OS) [47]. Thus, the introduction and application of reliable molecular techniques to the early detection and quantitation of *TP53*-, *ATM*- and *BIRC3*-deletions would improve the predictive and prognostic opportunities in CLL patients. The introduction of multiplex diagnostic ddPCR panels would reduce considerably the time required to produce a complete report.

### 7.2. Glioma and Glioblastoma

Gliomas are the most common tumor of the brain in humans with an incidence rate of 6.03 per 100,000 individuals every year [61].

Low-grade gliomas (LGGs) have an indolent course but may evolve to aggressive, high-grade gliomas (HGG, like glioblastomas) [62]. Glioblastoma multiforme (GBM) is an aggressive form of tumor of the central nervous system characterized by therapy resistance insurgence associated to the disease recurrence. The main treatment options are represented by surgery, radiotherapy and chemotherapy even though the prognosis remains dismal [63].

Gliomas can be classified into subtypes based on distinct molecular signatures. These molecular subtypes are characterized by different prognoses and treatment responses [64,65,66]. The molecular signatures allow the identification of epidermal growth factor receptor (*EGFR)* amplification, *TP53* and cyclin dependent kinase Inhibitor 2A (*CDKN2A*) deletion as associated to poor prognosis [67,68,69,70].

Meanwhile, other markers such as *IDH1* and *IDH2* gene mutations were correlated with a favorable prognosis [71].

The *EGFR* gene, given its correlation to a poor prognosis, is an important analytical marker in gliomas. *EGFR* is a transmembrane receptor tyrosine kinase that can initiate several intracellular signaling pathways contributing to cancer cell proliferation, inhibition of apoptosis, invasion, metastasis and stimulation of neovascularization [116,117]. The most frequent *EGFR* alterations are at genetic level (amplification or deletion like epidermal growth factor receptor variant III (EGFRvIII)) or at protein level (overexpression). *EGFR* alterations promote proliferation, angiogenesis and invasion and in glioblastoma also resistance to radiotherapy and chemotherapy [72,73,74].

EGFR protein overexpression is mainly studied by IHC whereas *EGFR* gene amplification preferentially by FISH [75]. The FISH assay is based on the *EGFR/CEP7* ratio. A probe hybridizes with chromosomal 7p12 region containing *EGFR* locus while a second probe hybridizes to the centromeric region 7p11.1. This latter one is used to count the number of copies of chromosome 7 [76,77]. The number of signals corresponding to the *EGFR* specific probe is directly related to the number of copies of this gene. The number of signals corresponding to the *CEP7* is directly related to the number of copies of chromosome 7. Normally, one probe is red and one is green. Yellow signals correspond to the overlap between red and green labeling. *EGFR* amplification is recorded when more than 10% of analyzed cells show an *EGFR* to *CEP7* ratio ≥2 [76,78].

*EGFR* abnormalities in brain tumor tissue can be also assessed by MLPA and ddPCR [79,80]. FISH and MLPA are widely used for diagnostic and research purposes. On the contrary, fewer articles exist describing ddPCR approach in glioma. Most of them are focused on *EGFR* mutations.

As mentioned before, *EGFR* is a prognostic marker that has to be combined with other molecular markers in order to identify different glioma subtypes. As an example, the codeletion of chromosomes 1p and 19q is strongly associated to oligodendroglioma [66,80,81,82]. Likewise, the fusion gene *KIAA1549:BRAF* is a specific molecular marker of the group of pilocytic astrocytoma [80,83,84].

### 7.3. Breast Cancer, Lung Cancer and Colorectal Cancer

Breast cancer, lung cancer and colorectal cancer are three examples of solid and often aggressive tumors. The investigation of specific gene alterations is useful for diagnostic and prognostic purposes.

Amplification and overexpression of *HER2* are associated with poor prognosis in breast cancer and suitable for anti-HER2 therapy. Thus, a correct identification of patients with *HER2* amplification is crucial. There are several methods to determine *HER2* status, including FISH, MLPA and ddPCR. FISH analysis usually uses a probe to the *HER2* gene and another for *CEP17*, as reference control. Patients with increased *HER2* along with increased *CEP17* copy numbers might be misclassified as non-amplified *HER2* as during polysomy of chromosome 17. Although several studies have reported that true polysomy of chromosome 17 is rare specific reporting guidelines have recently been developed to overcome this issue [96,97,98,99]. *HER2* and *c-Myc* gene amplification also were detected by MLPA and FISH in tissue samples of invasive breast cancer [101,102].

Moerland E. and coworkers evidenced a good correlation between MLPA, FISH and chromogenic in situ hybridization (CISH). However, the use of these different methods raises the issue of intra- and inter-laboratory reproducibility. Fixation time in FISH, together with other preanalytic variables, is another parameter affecting this approach [97]. Absolute *HER2* copy number can be determined by ddPCR without the need for calibration. So, ddPCR technique may be useful as an alternative to FISH and MLPA in *HER2* diagnosis [103].

Colorectal cancer (CRC) is one of the most deadly cancer in the world together with lung, liver and stomach cancer [114]. Advances in elucidating the molecular biology of these deadly cancers or other cancers led to the identification of a number of potential biomarkers that could be relevant in the clinical management of patients [115].

In CRC the main gene with copy number variation related to the disease is *EGFR*. The pivotal role of *EGFR* in cancer progression is used in the treatment of *RAS* wild-type metastatic CRC by EGFR-targeting monoclonal antibodies cetuximab and panitumumab [117,118,119,120,121,122,123]. *EGFR* is routinely analyzed by FISH. Likewise, *HER2* and *EGFR* copy number is evaluated by *EGFR/CEP7* ratio and can be affected by intra- and inter-laboratories variability [124,125,126]. The study of *EGFR* with other techniques such as MLPA and ddPCR are few and concern gene mutations instead of copy number variations.

The mutations in the rat sarcoma (*RAS*) gene are described more frequently than *EGFR* in CRC. The literature reports several studies on *RAS* using the three techniques that we are comparing in this review.

*RAS* gene family includes Kirsten rat sarcoma viral oncogene homolog (*KRAS*) and neuroblastoma RAS viral oncogene homolog (*NRAS*). It has also been suggested that *NRAS* mutations play a role in prognosis since patients harboring *NRAS* mutations have a significantly shorter survival compared to those bearing wild type *NRAS*. This is due to the fact that when *RAS* family members are mutated, the cells have an increased potential of invasion and metastasis [127,128,129,130].

As mentioned above, another leading cause of cancer-related death in the world is lung cancer. The clinical importance of the molecular phenotype of lung cancer bears therapeutic implications since different subtypes may respond to different targeted treatments.

*ALK* rearrangement represents a molecular target in a subset of non-small cell lung cancers (NSCLCs) [142]. Approximately 3%–7% of NSCLC harbor an *ALK* fusion gene, thus defining a tumor group that may be responsive to targeted therapy [143]. Fusion of *ALK* with the upstream partner *EML4* was found in NSCLC in 2007. This fusion is due to a chromosomal inversion on chromosome 2p, resulting in the formation of *EML4-ALK* fusion oncogene. Notably, *ALK* rearrangements occur almost exclusively in adenocarcinomas [144,145]. *ALK*-rearranged lung cancer is a unique molecular subgroup with a high sensitivity to *ALK* inhibitors and mutually exclusive with other well-known oncogenic mutations (*EGFR* or *KRAS*) [146,147].

*ALK*-rearranged lung cancer can be identified by immunohistochemistry (IHC), FISH or qRT-PCR with each method having advantages and disadvantages for screening. In 2011 *ALK* FISH was approved as a diagnostic kit for NSCLC [1,148,149,150]. However, FISH requires specialized technical resources and expertise, and the signals rapidly fade over time. Therefore, there has been interest in the development of other methods/technologies such as IHC, qRT-PCR and, in recent years, also NGS. Prior to its approval as a companion diagnostic kit for testing *ALK* rearrangement in NSCLC, the *ALK* FISH test was already being challenged by IHC. However, discrepancies in results obtained by FISH and IHC, such as *ALK* FISH+/*ALK* IHC− or *ALK* FISH−/*ALK* IHC+, have been reported in some cases [1,151,152,153,154,155].

In the past decade, due to new and better anti-ALK antibodies that detect *ALK* gene fusion, and automation of IHC procedures, a new generation *ALK* IHC has been developed and is considered as sensitive as *ALK* FISH for detecting this rearrangement [156,157,158,159]. Considering FISH, a gold standard reference, sensitivity and specificity of IHC are 90.0 and 97.8%, respectively [151].

Quantitative RT-PCR (qRT-PCR) has also been applied successfully to the detection of *EML4-ALK* fusion at the RNA level [160,161]. qRT-PCR is used to examine expression levels of the 5′ and 3′ portions of *ALK* transcripts, because the breakpoint in *ALK* consistently occurs at exon 20 and *EML4* (or other fusion partners). The resulting strong expression of ALK kinase domain leads to an unbalanced expression in 5′ and 3′ portions of *ALK* transcripts [143,162]. qRT-PCR could define *ALK* fusion partners and variants and also could be a suitable method for detecting *ALK* translocations using cytology samples from patients with primary lung cancer, especially when tissue samples are not available [161,162]. According to these studies, the concordances between qRT-PCR and *ALK* IHC or *ALK* FISH are approximately 95%-100% [160,162].

NGS-based methods are also becoming widely used in the detection of *ALK* rearrangements. The utility of these NGS-based methods emerged in the identification of *ALK* rearrangement-positive cases with an atypical *ALK* FISH signal pattern, even in cases that resulted in being negative by *ALK* FISH [9,163,164,165].

Finally, also ddPCR based assays have proven to be appropriate for the detection of *ALK* rearrangements. A significant advantage of ddPCR is the ability to detect and quantitate three variants of the *EML4-ALK* rearrangements that FISH cannot distinguish. Further advantages of ddPCR over FISH are represented by a lower limit of detection (LOD) associated to a lower cost of the test [166,167,168].

## 8. Conclusions

Molecular and cytogenetic techniques are the key to the study and diagnosis of cancer. For this purpose, some of their main applications are copy number variation analysis (deletion and duplication) and genetic rearrangements. Over the years, many targets have been successfully detected through FISH, MLPA and ddPCR (please, see Table 1).

In this article, we compared these three techniques evidencing the potential and characteristics of each one for cancer diagnostics.

Although FISH is often the golden standard for many targets, MLPA and ddPCR, according to the published data, can be much more sensitive and accurate. MLPA and ddPCR, however, differ in timing so that ddPCR bears a great potential for future applications.

## Figures and Tables

**Table 1:** Molecular targets investigated by the three techniques FISH, MLPA and ddPCR in the type of tumors described in the text.

Cancer Type/Subtype	FISH	MLPA	ddPCR	References
**Chronic Lymphocytic leukaemia**	- PBX1 - TCF3 - PDGFRB - TCRB - PAX5 - CDKN2A - ABL - BCR - ATM - TP53 - KMT2A - ETV6 - RUNX1 - DLEU1 - D13S25 - D12Z3 - TCRA/D - IGH - CRLF2 - MLL - TCF3/PBX1 - CKS1B - CDKN2C - CDKN2B - MYC - D20S108 - BCL2 - PML-RARα - 17p - 11q - 13q - BCR-ABL1 - FGFR1 - TCL1 - 14q32 - MYD88 - 9q11 - 6q - RB1 - IKZF1 - MYB - BTG1 - ETV6 - RUNX1 - p16 - E2A - TEL/AML1 - MLL - iAMP21 - KMT2A - SH2B3	- IKZF1 - TP53 - ATM - NOTCH1 - FBXW7 - PHF6 - RUNX1 - NRAS - DNMT3A - MAP3K7 - CDKN2A/2B - NF1 - SUZ12 - JAK1/2 - NUP214-ABL1 - MTAP - LEF1 - PTPN2 - HOX11L2 - PHF6 - iAMP21 - ETV6/RUNX1 - PAX5 - VPREB1 - KDM6A - STAG2 - 1q - 8q - 17q - 6q - EBF1	- PSMB6 - PGGT1B - UBQLN2 - UQCR2 - BCR/ABL - NOTCH1 - TP53 - SF3B1 - POT1 - XPO1 - SAMHD1 - CHD2 - FAM50A - MYD88 - NXF1 - ZMYM3 - APT10A - ATRX - EGR2 - FAT3 - IRF4 - FBXW7 - BIRC3	[7,43,47,48,49,50,51,52,53,54,55,56,57,58,59,60]
**Celebral cancer (Gliomas, glioblastoma)**	- EGFR - PDGFRA - TOP2A - RB1 - CDKN2A - CDKN2 - 1p36 - 1q21 - CEP7 - CEP10 - 19q13 - 19p13 - TP53 - PTEN - 9p21 - BRAF - EWSR1 - KIAA1549 - MYC - MYB - MYCC - MYCN - DMBT1 - p16 - ATRX - IDH1/2 - H3K27M - MIB1 - KLC1-ROS1 - TIMP1 - MET - NF1 e 2 - DAL-1 - HER2 - REST - 13q14	- ALK - PDGFRA - VEGFR2/KDR - EGFR - EGFRvIII - MET - FGFR1 - IDH1 e 2 - PTEN - MVT - 1p - 19q - MDM2 e 4 - CDKN2A - AKT1 - AURKA - BCAR2 - BCL2A1 - BCL2L1, 11, 13 - BCL6 - BCLG - BIRC1-5 - BRAF - BRMS1 - CCNA1 - CCND1-2 - CCNE1 - CDK4 -6 - CENPF - CYP27B1 - EMS1 - ERBB2-4 - ESR1 - EVI1 - FGF3-4 - FLJ20517 - GNAS - GSTP1 - HMGA1 - IGF1, 4, 5 - IRS2 - JAK2 - MET - MMP7 - MOS - MYCL1 - MYBL1-2 - MYC - MYCN - NFKBIE - NRAS - NTRK1-3	- MYD88 - IDH1 - IDH2 - TP53 - TERT - ATRX - H3F3A - HIST1H3B - BRAF - KIAA1549 - H3K27M - FGFR1 - EGFR	[61,62,63,64,65,66,67,68,69,70,71,72,73,74,75,76,77,78,79,80,81,82,83,84,85,86,87,88,89,90,91,92,93,94,95]
**Breast cancer**	- HER2 - MYC - CCND1 - MTDH - ETS - 12q14-q15 - 17q21-q25 - 20q13 - 11q13-q15 - 17q23.2-q23.3 - 20q13.12 - 3p21.32-p12.3 - EML4 - ALK - TOP2A - HER3 - EGFR - ESR1 - HER2	- HER2 - ZNF703 - FGFR1 - ADAM9 - IKBKB - MYC - CCND1 - C11ORF30 - CPD - MED1 - ERBB2 - CDC6 - TOP2A - MAPT	- HER2 - ESR1 - PGR - PUM1 - TERTp - TK1 - CDK4 - CDK6 - CDK9 - PIK3CA - PTPN2 - LRIG1	[96,97,98,99,100,101,102,103,104,105,106,107,108,109,110,111,112,113]
**Colorectal cancer**	- ADAMTS4 - KDM1A/LSD1 - PTEN - MALAT1 - BRAF - NRAS - KRAS - FGFR - SNHG1 - COX2 - PIK3CA - APC - CLIC1 - EGFR - MYC - CCND1 - CDX2 - CDH1 - TP53 - HER2/ERBB2 - SMAD7 - SMAD4 - ZNF217 - SLCO4A1-AS1 - DLEU1 - SMARCA1 - miR181 - MET - CCAT2 - NOMO-1 - TOP2A - CRY1 - CRY2 - NTRK1 - LMNA-NTRK1 - AXL - TOP1 - SOX2 - CDX2 - RAF - MLH1 - MSH2 - MUTYH - MSH6 - PMS2 - ALK - ROS1	- MLH1 - MSH2 - EPCAM - APC - 8p - 15q - 17p - 18q - 8q - 13q - 20q - MUTYH - MGMT	- BRAF - KRAS - Per17 - Per2 - Bmal1 - Clock - RAS - RAF - EGFR - GAEC1 - ITGA6	[114,115,116,117,118,119,120,121,122,123,124,125,126,127,128,129,130,131,132,133,134,135,136,137,138,139,140,141]
**Lung cancer**	- ALK - EML4 - EGFR - KRAS - ROS1 - TPM3 - SDC4 - SLC34A2 - CD74 - EZR - MET - KIF5B - TFG - NTRX - BRAF - RET	- ALK - RET - ROS - ERBB2 - MET - EGFR - BRAF - FGFR1 - MYC - CCND1 - CCND2 - CDK4 - CDK6 - MDM2 - MDM4	- ALK - EML4 - EGFR - KRAS - ROS1 - BRAF	[1,9,142,143,144,145,146,147,148,149,150,151,152,153,154,155,156,157,158,159,160,161,162,163,164,165,166,167,168,169,170,171,172,173,174]

## Data Availability

Not applicable.
